# Impact of point-of-care ultrasound on clinical decision-making at an urban emergency department in Tanzania

**DOI:** 10.1371/journal.pone.0194774

**Published:** 2018-04-25

**Authors:** Teri Ann Reynolds, Stas Amato, Irene Kulola, Chuan-Jay Jeffrey Chen, Juma Mfinanga, Hendry Robert Sawe

**Affiliations:** 1 Emergency Medicine Department, Muhimbili National Hospital, Dar es Salaam, Tanzania; 2 Emergency Medicine Department, Muhimbili University of Health and Allied Sciences, Dar es Salaam, Tanzania; 3 Department of Emergency Medicine and Global Health Sciences, University of California San Francisco, San Francisco, California, United States of America; 4 School of Medicine and Dentistry, University of Rochester, Rochester, New York, United States of America; 5 School of Medicine, University of California San Francisco, San Francisco, California, United States of America; Yokohama City University, JAPAN

## Abstract

**Background:**

Point of care ultrasound (PoCUS) is an efficient, inexpensive, safe, and portable imaging modality that can be particularly useful in resource-limited settings. However, its impact on clinical decision making in such settings has not been well studied. The objective of this study is to describe the utilization and impact of PoCUS on clinical decision making at an urban emergency department in Dar es Salaam, Tanzania.

**Methods:**

This was a prospective descriptive cross-sectional study of patients receiving PoCUS at Muhimbili National Hospital’s Emergency Medical Department (MNH EMD). Data on PoCUS studies during a period of 10 months at MNH EMD was collected on consecutive patients during periods when research assistants were available. Data collected included patient age and sex, indications for ultrasound, findings, interpretations, and provider-reported diagnostic impression and disposition plan before and after PoCUS. Descriptive statistics, including medians and interquartile ranges, and counts and percentages, are reported. Pearson chi squared tests and p-values were used to evaluate categorical data for significant differences.

**Results:**

PoCUS data was collected for 986 studies performed on 784 patients. Median patient age was 32 years; 56% of patients were male. Top indications for PoCUS included trauma, respiratory presentations, and abdomino-pelvic pain. The most frequent study types performed were eFAST, cardiac, and obstetric or gynaecologic studies. Overall, clinicians reported that the use of PoCUS changed either diagnostic impression or disposition plan in 29% of all cases. Rates of change in diagnostic impression or disposition plan increased to 45% in patients for whom more than one PoCUS study type was performed.

**Conclusions:**

In resource-limited emergency care settings, PoCUS can be utilized for a wide range of indications and has substantial impact on clinical decision making, especially when more than one study type is performed.

## Introduction

Point-of-care ultrasound (PoCUS) has been shown to improve diagnostic accuracy;[1–10] facilitate faster diagnosis, consultation and definitive treatment;[10–19] and decrease complication rates when used for procedural guidance.[20–23] PoCUS is affordable and cost-effective,[19,22–25] has been shown to reduce the use of more invasive and expensive diagnostic modalities,[24–27] and has been shown to be effective when performed by a variety of clinical providers in a range of clinical settings.[27–36]

All of these qualities make PoCUS particularly suited for resource-limited settings where other imaging modalities may be unavailable, impractical, or expensive, and patient transfer for radiology services may be burdensome or impossible. There has been a substantial increase in the use of PoCUS in resource-limited settings over the past decade,[11,34–54] but there is limited information on the impact of PoCUS on clinical decision-making in such settings. In Rwanda, pilot studies have shown that training in bedside ultrasound is feasible in a rural district hospital and that its use changes diagnosis in over 40% of cases.[45] In Liberia, the temporary introduction of ultrasound changed diagnosis in 30% to 60% of cases, depending on indication.[51]

Muhimbili National Hospital (MNH), Tanzania’s largest national referral hospital and the main teaching hospital of the Muhimbili University of Health and Allied Sciences (MUHAS), opened the country’s first full capacity emergency department in January 2010, the Emergency Medical Department (EMD). The MNH EMD is the main intake area for medical and surgical patients at this national public referral hospital with an annual volume of approximately 40,000 patients per year at the time of this study.[55,56] The availability of radiology studies at MNH EMD is limited by cost, time of day, extended wait times, and by distance for unstable patients who cannot be transported out of the emergency care area. Radiology-performed ultrasound services are available at MNH, though often with substantial delay, which limits their utility for clinical decision-making in the emergency department. Computed tomography (CT) is available, though its accessibility can be limited by service interruptions, or by a requirement for pre-payment for some patients. The emergency medicine residency program at MNH has fully incorporated PoCUS training into its curriculum, and inservice training is provided for all registrar (non-specialist) doctors working in the EMD [50]. MNH EMD providers frequently perform PoCUS for a range of indications. Previous research at MNH has shown that temporary introduction of bedside ultrasound to a surgical service accelerated diagnostic workup, shortened hospitalization, and may have helped avoid laparotomy in some cases, but there has been no study of the utilization of PoCUS in the MNH emergency department setting.[48] We aimed to characterize the utilization and impact of PoCUS on clinical decision-making in the MNH EMD.

## Methods

This is a prospective descriptive cross-sectional study of PoCUS at MNH EMD. This study was approved by the institutional review boards of Muhimbili National Hospital and the University of California, San Francisco, and, given the anonymity of the data collected, informed consent was waived by both institutional review boards. A standardised data collection form was used by a research assistant on each ultrasound performed during times when research assistants were available in the department within a ten-month period (August 2014 to May 2015). Research assistants were trained to complete the data collection form, and collected responses from providers performing ultrasound examinations in real time. The availability of research assistants was determined entirely by other administrative staffing considerations and was not correlated with clinical staffing or patient volume. At the time of this study, EMD providers performing PoCUS included 12 specialist attending physicians, 10 resident physicians and 17 registrars. All ultrasound studies were performed with a SonoSite mTurbo (SonoSite Corp., Bothell, WA).

Providers followed specific protocols for each type of scan and reported on image adequacy, findings, and interpretation. The MNH EMD protocols were derived from existing university and professional society guidelines and the Partners in Health Manual for Ultrasound in Resource-Limited Settings.[57] For extended focused assessment with sonography for trauma (eFAST), views were obtained for the hepatorenal, pericardial, perisplenic, suprapubic, bilateral costophrenic angles, and bilateral lungs; providers indicated if free fluid and/or lung sliding were visualized. For thoracic scans, views were obtained of bilateral lungs, which were assessed for pleural fluid, b-lines, and/or parenchymal consolidations. For cardiac scans, providers obtained parasternal long, parasternal short, apical four chamber, subxiphoid four chamber, and subxiphoid long axis (IVC) views and assessed for pericardial effusion, global systolic function, chamber size, and IVC collapsibility. For abdominal scans, views were obtained of the aorta (both transverse and longitudinal) and/or the gallbladder (long and short axes), and providers assessed for abdominal aorta diameter, gallstones/sludge, gallbladder wall-thickening, pericholecystic fluid, and/or dilatation of the extrahepatic bile ducts. For OB/GYN scans, providers performed transabdominal and/or transvaginal scans and assessed for intrauterine pregnancy, adnexal masses, and/or fetal heart activity. For urologic/renal exams, both transverse and longitudinal views were obtained of the bladder and kidneys, which were assessed for hydronephrosis, calculi, and cysts/masses. For DVT scans, views were obtained of the proximal, middle, and distal femoral veins and the popliteal veins, which were assessed for compressibility. For soft tissue studies, scans assessed for fluid collections, evidence of peripheral oedema and evidence of fracture, as relevant. Other findings, such as intussusception, situs inversus and ocular findings, were occasionally reported in the free text ‘other’ section of the data form.

Case data collected included patient age and sex, indications for ultrasound, ultrasound findings, provider-reported diagnostic impression and disposition plan before and after ultrasound. This information was transcribed from handwritten forms and entered into Stata (version 14) for data analysis. Descriptive statistics including medians and interquartile ranges, and counts and percentages, are reported. Pearson chi squared tests were applied to categorical data and p-values were used to evaluate for significant differences.

We report on patient characteristics including age, sex, and proportion presenting for injury or pregnancy (Table 1). Frequency and proportions were tabulated for PoCUS indications and study types (Tables 2 and 3 respectively). Proportions are described to be out of total patients and/or out of total scans as relevant, since many patients underwent more than on PoCUS study. In particular, impact was measured based on reported change of diagnostic impression or disposition plan for each patient (Table 4), and proportions are reported relative to total patients. A Pearson chi squared test was utilized to examine whether there was a difference in impact on clinical decision-making between different study types and number of diagnostic study types (Tables 5 and 6 respectively). Frequency and proportions were tabulated for post-ultrasound diagnostic impressions which were coded and aggregated into categories based on Clinical Classification Software categories (Table 7).[58] Frequency and proportions of patient post-ultrasound disposition plans were tabulated (Table 8).

**Table 1:** Patient characteristics.

Patient Characteristics	
Median age, years (IQR)	32 (23–50)
Adult (> 18 years) % (n)^a^	79.3 (622/784)
Male % (n)^b^	55.8 (434/778)
Pregnant patients, as % of females (n)	16.6 (57/344)
Trauma patients, as % of total (n)	42.1 (330/784)
Patients with abnormal reported PoCUS findings % (n)	48.7 (382/784)

Characteristics of patients who underwent point of care ultrasound.

^a^ There is missing data for age on 55 (7%) patients.

^b^ There is missing data for sex on 6 (<1%) patients.

**Table 2:** Top indications for PoCUS.

Indications for Ultrasound^a^	n (%)	Abnormal Findings, n (%)
Trauma	330 (42.1)	62 (18.8)
Respiratory^b^	168 (21.4)	142 (84.5)
Abdomino-Pelvic Pain	114 (14.5)	77 (67.5)
Procedure	45 (5.7)	21 (46.7)
Abdominal Distension	41 (5.2)	31 (75.6)
Tachycardia	35 (4.5)	25 (71.4)
Pregnancy	33 (4.2)	27 (81.8)
Vaginal Bleeding	28 (3.6)	20 (71.4)
Chest Pain	26 (3.3)	18 (69.2)
Cardiac Arrest	22 (2.8)	19 (86.4)
Peripheral Oedema	20 (2.6)	17 (85.0)
Hypotension/Shock	20 (2.6)	17 (85.0)
Palpitations	19 (2.4)	14 (73.6)
Altered Mental Status	18 (2.3)	12 (66.7)
Decreased Urinary Output	11 (1.4)	9 (81.8)
Fever	11 (1.4)	7 (63.6)
Other Indications	59 (7.5)	41 (69.5)
Missing	24 (3.1)	12 (50.0)
Total	1024	571 (55.8)

Provider reported indications for point of care ultrasound.

^a^ More than one indication was reported in 173 (22.1%) patients.

^b^ Respiratory presentations included dyspnoea, orthopnoea, hypoxia, tachypnoea.

**Table 3:** Frequency of ultrasound study types.

PoCUS Type^a^	n (%)	Abnormal Findings, n (%)
eFAST	467 (59.6)	161 (34.5)
Cardiac	215 (27.4)	173 (80.5)
OB/GYN	79 (10.1)	58 (73.4)
Thoracic	64 (8.2)	54 (84.4)
Abdominal	57 (7.3)	40 (70.2)
Procedure	45 (4.6)	21 (46.7)
Renal	39 (5.0)	32 (82.1)
LE Doppler	10 (1.3)	8 (80.0)
Musculoskeletal	10 (1.3)	8 (80.0)
**Total**	986	534 (54.2)

Ultrasound study types utilized by providers and frequency of abnormal findings.

^a^ More than one ultrasound study type was utilized in 157 (20.0%) patients.

**Table 4:** Reported impact of ultrasound on clinical decision-making.

Impact on Clinical Decision-Making	% of Patients (n)^a^	CI (95%)
Change in Diagnostic Impression	27.0 (203)	23.9–30.4
Change in Disposition Plan	13.1 (99)	10.8–15.8
Change in Diagnostic Impression or Disposition Plan	28.8 (217)	25.6–32.2

Impact of point of care ultrasound on clinical decision-making reported as change in diagnostic impression or change in disposition plan.

^a^ There is missing data for diagnosis and disposition on 8 (1%) of patients.

**Table 5:** Change in diagnostic impression or disposition plan by study type.

	All patients (n = 753)		Patients with 1 study type (n = 614)		Patients with > 1 study type^a^ (n = 139)	
Study Type	Change in Dx or Dispo % (n/total)	Chi2 (p)	Change in Dx or Dispo % (n/total)	Chi2 (p)	Change in Dx or Dispo % (n/total)	Chi2 (p)
Renal	46.2 (18/39)	6.026 (0.014)*	38.1 (8/21)	1.960 (0.162)	55.6 (10/18)	0.874 (0.350)
Thoracic	45.2 (28/62)	8.797 (0.003)*	33.3 (4/12)	0.444 (0.505)	48.0 (24/50)	0.226 (0.635)
OB/GYN	39.0 (30/77)	4.302 (0.038)	34.5 (19/55)	2.880 (0.090)	50.0 (11/22)	0.231 (0.631)
Musculoskeletal	30.0 (3/10)	0.007 (0.934)	25.0 (2/8)	0.000 (0.996)	50.0 (1/2)	0.018 (0.984)
LE Doppler	30.0 (3/10)	0.007 (0.934)	25.0 (2/8)	0.000 (0.996)	50.0 (1/2)	0.018 (0.984)
eFAST	29.7 (137/461)	0.469 (0.493)	25.1 (89/354)	0.002 (0.968)	44.9 (48/107)	0.040 (0.841)
Cardiac	29.1 (62/213)	0.012 (0.912)	19.7 (26/132)	2.594 (0.107)	44.4 (36/81)	0.061 (0.806)
Abdominal	26.8 (15/56)	0.122 (0.727)	16.7 (4/24)	0.941 (0.332)	34.4 (11/32)	2.011 (0.156)
**Total**	28.8 (217/753)		25.1 (154/614)		45.3 (63/139)	

Impact of point of care ultrasound on clinical decision-making by study type.

^a^ Among patients with recorded diagnosis and disposition, there were 139 patients with greater than one study type.

**Table 6:** Change in diagnostic impression or disposition plan by number of diagnostic studies.

Number of PoCUS Studies	% of Patients with Change in Diagnosis or Disposition (n/total)	CI (95%)	Chi^2^ (p)
1	25.1 (154/614)	21.7–28.7	22.640 (0.000)
2	45.2 (47/104)	35.4–55.3	15.771 (0.000)
3	47.1 (16/34)	29.8–64.9	5.776 (0.016)
4	0.0 (0/1)	0–97.5	0.405 (0.524)
Total	28.8 (217/753)		

Impact on clinical decision-making by number of diagnostic studies.

**Table 7:** Post-ultrasound provider-reported diagnostic impressions.

Diagnostic Impression	n	%
**Trauma**	**330**	**42.1**
Intracranial injury	134	17.1
Extremity fracture(s)	76	9.7
Other fracture(s)	23	2.9
Other trauma	112	14.3
**Cardiovascular**	**157**	**20.0**
Heart failure	70	8.9
Cardiomyopathy	19	2.4
Pericardial effusion	18	2.3
Hypertension	13	1.7
Arrhythmia	7	0.9
Valve disease	6	0.8
Venous thrombosis or embolism	6	0.8
Aortic aneurysm or dissection	5	0.6
Other cardiovascular	39	5.0
**Abdomino-pelvic**	**112**	**14.3**
Renal failure	19	2.4
Other renal and urinary tract disease	21	2.7
Hepatobiliary disease	14	1.8
Female reproductive disease	6	0.8
Other abdomino-pelvic	50	6.4
**Pregnancy**	**67**	**8.5**
Ectopic pregnancy	18	2.3
Spontaneous abortion	15	1.9
Other pregnancy related complications	35	4.5
**Infectious**	**58**	**7.4**
Tuberculosis	13	1.7
Septicemia	12	1.5
Pneumonia	11	1.4
Peritonitis	8	1.0
Meningitis	3	0.4
Other infectious	19	2.4
**Pulmonary**	**53**	**6.8**
Pulmonary edema	18	2.3
Pleural effusion	18	2.3
Pneumothorax	9	1.1
Hemothorax	5	0.6
Asthma or COPD	4	0.5
Pulmonary embolism	1	0.1
**Neoplasms**	**22**	**2.8**
**Death determination**	**21**	**2.7**
**Other**	**17**	**2.2**
**Missing**	**33**	**4.2**

Provider-reported diagnostic impressions after ultrasound.

**Table 8:** Post-ultrasound disposition plans.

Disposition Plans	n	%	CI (95%)
Muhimbili Orthopeadic Institute^a^	260	34.7	29.9–36.6
Cardiology	112	14.9	11.9–16.9
General Medical	102	13.6	10.7–15.6
General Surgery	67	8.9	6.7–10.7
Obstetrics/Gynecology	66	8.8	6.6–10.6
Operating Theatre	37	4.9	3.4–6.4
Discharge	33	4.4	2.9–5.9
Pediatric	26	3.5	2.2–4.8
Surgical Sub-Specialty	26	3.5	2.2–4.8
Mortuary	21	2.8	1.7–4.1
Intensive Care Unit	4	0.5	0.1–1.3
Burn	1	0.1	0.0–0.7
Ophthalmology	1	0.1	0.0–0.7
Missing	34	4.3	

Provider-reported disposition plans after ultrasound

^a^The Muhimbili Orthopaedic Institute (MOI) is a semi-independent institute on the MNH campus providing orthopaedic and neurosurgical services.

## Results

### Patient characteristics

Data were collected on 986 studies performed on 784 patients. The majority of patients (79%) were adults over the age of 18 years, and the median patient age was 32 (Table 1). There were 55 patients (7%) for whom age was not recorded. Six patients (<1%) were missing documentation of sex. Among patients with documented sex, 55.8% were male. Of the female patients, 16.6% were pregnant by history or testing. Trauma patients made up 42% of this sample, and of the trauma patients, 73% were male.

### Ultrasound indications

Providers reported a clinical indication for ultrasound in 97% of patients. More than one indication was reported in 22.1% of patients overall. The top indications for ultrasound were trauma, (non-traumatic) respiratory presentations, and (non-traumatic) abdomino-pelvic pain (Table 2). Patient indications that most commonly associated with abnormal ultrasound findings included hypotension/shock (85%), peripheral oedema (85%), and respiratory complaints (84.5%).

### Ultrasound study type

Including the use of PoCUS for procedures, 157 (20.0%) patients had more than one type of ultrasound study. The most frequent ultrasound study types were eFAST, cardiac and obstetric/gynaecologic (Table 3). Study types that were most commonly associated with abnormal ultrasound findings included thoracic (84.4%), renal (82.1%) and cardiac (80.5%).

### Ultrasound guided procedures

A total of 45 patients, 5.7% of this sample, underwent one or more ultrasound-guided procedures. Twenty-three of these patients underwent only procedural ultrasound for central or peripheral vascular access and are not included in the denominator for Tables 4, 5 and 6 on impact. The most frequent procedures were central venous access (23) and peripheral venous access (7). Other ultrasound-guided procedures included thoracentesis (5), paracentesis (4), pericardiocentesis (3), and thoracostomy (2).

### Impact on clinical decision-making

Of the 761 patients that underwent a diagnostic ultrasound study, clinician-reported diagnostic impressions changed in 27% and disposition plans changed in 13% of patients after ultrasound. A change in either diagnostic impression or disposition plan was reported for 28.8% patients overall (95% CI: 25.6–32.2%). Eight patients (1%) had missing data for diagnosis and disposition and are not included in the denominator for Tables 4 and 5.

When only one ultrasound study was utilized, there were no significant differences in impact on clinical decision-making among study types (see Table 5). Among all patients, including those for whom more than one ultrasound study was performed, renal and thoracic studies were found to have significantly higher rates of impact than other study types.

Rates of reported change in diagnostic impression or disposition plan following ultrasound significantly increased with the number of studies performed (Table 6). When providers performed only one PoCUS study type, they reported a change in diagnostic impression or disposition plan in 25.1% of cases. Among patients for whom two PoCUS study types were performed, providers reported a change in either diagnostic impression or disposition plan in 45.2% of cases, and among patients for whom three PoCUS studies were performed, providers reported a change in either diagnostic impression or disposition plan in 47.1% of cases. Overall, in patients for whom greater than one PoCUS study was performed, there was a 45.3% rate of change in diagnosis and disposition.

### Post-ultrasound diagnostic impressions and disposition plans

Post-ultrasound diagnostic impressions and disposition plans are displayed in Tables 7 and 8. Diagnostic impressions were coded based on the Clinical Classification Software[58] categories and aggregated into categories of trauma (42.1%), cardiovascular (20.0%), abdomino-pelic (14.3%), pregnancy (8.5%), infectious (7.4%), pulmonary (6.8%), neoplasm (2.8%), and determination of death (2.7%). Multiple diagnoses were recorded for 6.8% of patients.

The most common disposition plan was to Muhimbili Orthopaedic Institute (MOI), a semi-independent institute within MNH, which provides orthopaedic and neurosurgical services. Other common disposition plans included cardiology, general medical, general surgical, and obstetric and gynaecologic units. Six patients (0.8%) had multiple dispositions reported (Table 8).

## Discussion

As ultrasound technology has become more portable and affordable, PoCUS has shown increasing promise in low-resource settings as a reliable tool for both diagnosis and treatment.[20–23] This study describes 986 scans performed for 784 patients seen at the emergency department of the largest public referral and teaching hospital in Tanzania. Over 50 distinct indications for ultrasound were recorded. The top indication for ultrasound was trauma, followed by respiratory presentations and abdomino-pelvic pain.

There was a high rate of utilization of ultrasound for eFAST, which accounted for over half the scans performed in this study. Cardiac, obstetric or gynecologic, thoracic, abdominal, and renal studies were also common, though prior studies have found higher relative rates of utilization for obstetric and gynecologic applications. These studies were conducted in smaller rural hospitals (Rwanda) or among inpatients (Liberia), rather than at a dedicated emergency department in an urban center, which may have influenced the relative prevalence of acute trauma in our study population.[45,51] In addition, many pregnant patients presenting acutely to Muhimbili are evaluated directly by obstetric services, rather than being assessed and treated in the emergency department, and this likely influenced our results.

PoCUS had a substantial impact on patient care, changing diagnostic impression or disposition plan in 28.8% of cases overall. While all study types had impact on clinical decision-making, renal and thoracic studies had the highest. The overall impact on clinical decision-making in this study is lower than previous studies in Sub-Saharan Africa, which may be attributable to the differences in presenting complaints or provider skill, MNH’s status as a referral facility (such that many patients present with some diagnostic results in hand), or the relative availability of other resources to support diagnosis. In our study, similar to prior studies, change in disposition plan was reported less frequently than change in diagnostic impression.

Interestingly, PoCUS led to significantly more changes in diagnostic impression or disposition plan (up to 47%) among patients undergoing multiple studies. This pattern spans all study types and may suggest that providers perform more ultrasound studies in patients for whom there is greater diagnostic uncertainly, and/or that clinical data from additional PoCUS studies helps providers identify new or additional diagnoses.

## Limitations

This study has several limitations. First, the study is based on provider-reported data. While providers were asked to report the pre-ultrasound diagnostic impression prior to performing the ultrasound, we could not ensure that this protocol was followed in all cases. This effect would, however, be expected to underestimate the actual impact of PoCUS (if we assume that providers are more likely to report a ‘pre’-ultrasound diagnostic impression consistent with ultrasound findings), so our results should represent at least a minimum level of impact. In addition, data were recorded in real time during the clinical encounter, which should mitigate recall bias and increase accurate reporting. Because research assistants were not available to record ultrasound impressions at all times, this study may not accurately characterize all ultrasound utilization at the site. However, this is mitigated by the enrolment of consecutive patients during periods in which research assistants were available.

We did not, however, verify the quality or accuracy of the ultrasound studies and interpretations; we report only on the relationship between reported findings and reported diagnoses/dispositions. Ultrasound is a highly operator-dependent imaging modality, and while there are ongoing quality control efforts at the site comparing the provider interpretation of saved studies with those of independent evaluators, this study does not provide any information on the accuracy of provider diagnoses or quality of ultrasound studies performed.

Finally, there were some missing data for some patients. Based on discussions with providers and research staff, this most often occurred due to high patient volume and limited time to complete the form. The impact of this, however, is unlikely to reflect systematic bias, and its impact should in any case be limited, as missing data occurred at a maximum rate of 7% (age of patient). In order to allow readers to assess the importance of this limitation, we have identified the rate of missing data in text for each section of results.

## Conclusion

This study demonstrates the impact of PoCUS on clinical decision-making at a public urban emergency department in East Africa. PoCUS substantially impacted clinicians’ decisions on diagnostic impressions and, less often, on disposition plans. More studies are needed to evaluate the quality accuracy of PoCUS, and the impact of PoCUS on clinical interventions and outcomes, in such settings. Overall, this study contributes to a longitudinal understanding of the evolving implementation and utilization of PoCUS in the region.
